# Prevalence of multi-drug resistant, extensively drug resistant, pan drug resistant bacteria from intensive care units of a tertiary care teaching hospital, Amalapuram, India

**DOI:** 10.6026/973206300211475

**Published:** 2025-06-30

**Authors:** Padmaja N., Deborah Purushottam M., Srilalitha V.

**Affiliations:** 1 Department of Microbiology, Konaseema Institute of Medical Sciences and Research Foundation, Amalapuram, Andhra Pradesh, India

**Keywords:** Multidrug-resistant, *gram-negative bacilli*, antibiotic susceptibility

## Abstract

The prevalence of MDR, XDR and PDR gram-negative bacteria, drug resistance and sensitivity patterns in the intensive care unit of the tertiary care hospital Amalapuram from June 2023 to July 2024 is of interest. The study included all patients who were admitted to the intensive care unit and various clinical samples like blood, urine, respiratory samples, body fluids and pus received in the lab were inoculated on different media like Mac-Conkey agar and blood agar according to Standard Microbiological methods. Bacterial identification was done based on colony morphology, Gram stain and various biochemical reactions after obtaining pure growth. Gram-negative bacteria were identified using a series of biochemical tests, like oxidase, catalase, indole production, methyl red, V-P, citrate, urease and triple sugar iron agar tests. Antibiotic susceptibility testing was performed using the Kirby-Bauer disk diffusion method according to CLSI guidelines. We found a high frequency of antibiotic resistance in ICU isolates, primarily caused by *gram-negative bacilli* like *Klebsiella* and *E. coli*.

## Background:

The recent discovery of antimicrobials represents an essential turning point in medicine [1]. Antimicrobials are natural or synthetic compounds that, at particular concentrations, prevent bacterial growth [2]. Antibiotics are the most often administered medications in the Intensive Care Unit [3]. The use of appropriate antimicrobial agents is essential to effectively treat life-threatening conditions that result in sepsis and septic shock [4]. Before the emergence of antibiotic resistance, they served as effective therapies for infectious diseases. The development of antibiotic-resistant microorganisms may result from the repetitive and improper application of particular antimicrobial drugs within a hospital or healthcare environment [5]. Experts predicted that 20-50% of antibiotic usage was inappropriate [6]. Drug resistance has proliferated to such a degree that it has become a significant concern in the 21st-century healthcare system [1]. Antibiotic resistance is far more prevalent in developing nations like India than in high-income countries [7]. The World Health Organization (WHO) has recently acknowledged the emergence of multidrug-resistant bacterial species as a critical public health concern [8]. Multidrug resistance is the emergence of resistance to at least one antibiotic from three or more classes [9]. The prevalence of multidrug-resistant *gram-negative bacilli* is increasing rapidly. In developing nations, infections from multidrug-resistant gram-negative bacteria have led to markedly prolonged hospital stays, increased morbidity and elevated mortality rates over the past twenty years [10]. Gram-negative bacteria, including members of the Enterobacteriaceae family (*Escherichia coli*, *Klebsiella spp*., *Enterobacter spp*. and *Proteus spp*.) and non-lactose fermenters (*Pseudomonas spp*. and *Acinetobacter spp*.), are recognized as the principal agents of multidrug-resistant bacterial infections [11]. Drug-resistant disease mortality rates are anticipated to rise by 70% globally by 2050, leading to the deaths of 40 million people [12]. Antibiotic resistance in India constitutes a substantial public health hazard. India possesses one of the highest burdens of infectious diseases globally, with susceptibility patterns varying by region [2]. The significant variation in the prevalence of major pathogens and their antibiotic sensitivities, particularly in specific ICUs, highlights the importance of local susceptibility patterns for facilitating rational antimicrobial dosing and possibly delaying the emergence of antimicrobial resistance. The ICU environment significantly influences the development of drug resistance, as empirical antibiotic treatment is frequently initiated before the availability of culture and sensitivity results [13, 14]. It is essential to carefully select and manage antibiotic treatment to prevent the emergence of resistance. Therefore, it is of interest to assess the prevalence of MDR, XDR and PDR gram-negative bacteria, drug resistance and sensitivity patterns in the intensive care unit of the tertiary care hospital Amalapuram.

## Methodology:

## Study design:

This cross-sectional study was conducted at the Tertiary Care Teaching Hospital, KIMS Amalapuram, over twelve months following ethical approval from the Institutional Ethics Committee (IEC/CD/2024). Before inclusion in the study, written informed consent was obtained from all patients.

## Inclusion and exclusion criteria:

All patients admitted to the Intensive Care Unit (ICU), Medical ICU (MICU), Surgical ICU (SICU) and Cardiac ICU (CCIU) were included. Clinical samples were collected exclusively from patients hospitalized in these intensive care units between June 2023 and July 2024. Patients admitted to the Pediatric or Neonatal ICUs and those with suspected Gram-positive bacterial infections or fungal etiologies were excluded.

## Data collection procedure:

Clinical samples-including sputum, blood, urine, respiratory specimens, body fluids and pus-were collected from ICU patients and processed in the microbiology laboratory using standard microbiological techniques. Samples were inoculated onto MacConkey agar, blood agar and nutrient agar and incubated at 37°C for 24 hours. Bacterial growth was identified based on colony morphology, Gram staining and motility. Gram-negative bacteria were further identified using biochemical tests such as catalase, oxidase, indole production, methyl red, Voges-Proskauer, citrate utilization, urease and triple sugar iron agar tests. Gram-positive bacteria were identified based on Gram reaction, catalase test, hemolytic pattern, coagulase test and other relevant tests. Antibiotic susceptibility testing (AST) was performed using the Kirby-Bauer disk diffusion method on Mueller-Hinton agar by Clinical and Laboratory Standards Institute (CLSI) guidelines. For Gram-negative bacterial isolates, first-line antibiotics included amoxiclav, ampicillin-sulbactam, ceftazidime, ceftriaxone, levofloxacin, tetracycline, co-trimoxazole and gentamicin. Second-line antibiotics included piperacillin-tazobactam, tobramycin, cefoperazone-sulbactam and ceftazidime-avibactam. Meropenem and minocycline were used as reserved drugs. In urine samples, nitrofurantoin and norfloxacin were tested. For non-fermenting organisms such as *Pseudomonas*, *Acinetobacter*, *Burkholderia* and *Stenotrophomonas*, tobramycin was included as a second-line drug for AST.

## Data analysis and interpretation:

Data were analyzed using Microsoft Excel 2007 and the Statistical Package for the Social Sciences (SPSS®), version 24. Descriptive statistics were used to summarize patient demographics, organism isolation patterns and antibiograms. Microsoft Excel 2007 created graphic representations. Correlation analysis of antibiotic resistance patterns among bacterial isolates was conducted using the Chi-square and Pearson correlation tests. A p-value of less than 0.05 was considered statistically significant.

## Results:

In total, 3312 ICU samples were received, of which 1195 (36%) tested culture-positive. Among the culture-positive samples, 792 (66.3%) were *gram-negative bacilli* (GNB), 248 (20.8%) were *Gram-positive cocci* (GPC) and 155 (13%) were *Candida species* (Table 1 - see PDF). Of the 792 *gram-negative bacilli* (GNB) isolates, *Klebsiella* constituted the predominant group, accounting for 277 (34.9%) isolates. Among these, 59 (21.2%) exhibited resistance to at least one antibiotic, including 27 (45.7%) multidrug-resistant (MDR) isolates, 21 (35.5%) extensively drug-resistant (XDR) isolates and 11 (18.6%) pan drug-resistant (PDR) isolates. *E. coli* represented 244 (30.8%) of the isolates, with 55 (22.5%) exhibiting resistance, comprising 27 (49%) multidrug-resistant (MDR) isolates, 24 (43.6%) extensively drug-resistant (XDR) isolates and 4 (7.2%) pan drug-resistant (PDR) isolates. *Pseudomonas* exhibited 148 isolates (18.6%), of which 25 (16.8%) were resistant, comprising 11 (44%) multidrug-resistant (MDR) isolates, 10 (40%) extensively drug-resistant (XDR) isolates and 4 (16%) pan-drug-resistant (PDR) isolates. *Acinetobacter* accounted for 93 (11.7%) isolates, of which 27 (29%) were resistant, comprising 7 (25.9%) multidrug-resistant (MDR) isolates, 14 (51.8%) extensively drug-resistant (XDR) isolates and 6 (22.2%) pan drug-resistant (PDR) isolates. Enterobacter exhibited no resistance among 15 isolates (1.89%). Proteus exhibited 13 isolates (1.64%), with 2 (15.3%) isolates demonstrating resistance, both of which were multidrug-resistant (MDR). *Stenotrophomonas* and *Burkholderia*, each with one isolate (0.12%), exhibited complete XDR resistance (Table 2 - see PDF). The antibiotic susceptibility profile of *E. coli* isolates from urine samples demonstrated inadequate sensitivity (<60%) to first-line antibiotics, such as amoxiclav, ampicillin-sulbactam, cephalosporins, levofloxacin and gentamicin. Among second-line antibiotics, cotrimoxazole, piperacillin-tazobactam and ceftazidime-avibactam showed low sensitivity, whereas cefoperazone-sulbactam, other reserved agents such as carbapenems and minocycline exhibited moderate sensitivity (60-80%). Nitrofurantoin continued to demonstrate efficacy against *E. coli* isolates. *Klebsiella* isolates exhibited comparable resistance profiles, displaying limited sensitivity to most first-line and second-line antibiotics, except gentamicin and cefoperazone-sulbactam, which exhibited moderate sensitivity (60-80%). Carbapenems and minocycline exhibited moderate efficacy, whilst nitrofurantoin showed low sensitivity. Proteus isolates demonstrated inadequate sensitivity (<60%) to first-line antibiotics and cotrimoxazole. Piperacillin-tazobactam, ceftazidime-avibactam, cefoperazone-sulbactam and carbapenems exhibited intermediate sensitivity, ranging from 60% to 80%. Nitrofurantoin and norfloxacin proved ineffective. *Pseudomonas* isolates exhibited limited sensitivity to first-line antibiotics and specific second-line agents. Gentamicin, piperacillin-tazobactam and tobramycin exhibited moderate sensitivity (60-80%), but carbapenems had strong sensitivity (80%)-urinary agents such as nitrofurantoin and norfloxacin showed inadequate sensitivity. *Acinetobacter* isolates had the most significant resistance, demonstrating limited sensitivity to most first-line and second-line antibiotics. Gentamicin and minocycline exhibited intermediate sensitivity (60-80%), whereas tobramycin displayed the highest sensitivity (96%). Urinary antibiotics, such as nitrofurantoin and norfloxacin, demonstrated limited efficacy. Tobramycin revealed significant efficacy (≥80% sensitivity), specifically against *Acinetobacter* (96%). Moderate efficacy (60-80%) was observed with carbapenems and minocycline against *E. coli*, gentamicin and cefoperazone-sulbactam against *Klebsiella* and a combination of piperacillin-tazobactam, ceftazidime-avibactam and cefoperazone-sulbactam against Proteus. Pseudomonas exhibited intermediate sensitivity to gentamicin, piperacillin-tazobactam and tobramycin, whereas *Acinetobacter* had moderate responsiveness to gentamicin and minocycline. All bacteria revealed reduced susceptibility (<60%) to commonly used antibiotics, including amoxiclav, ampicillin-sulbactam, cephalosporins (1st to 3rd generation), levofloxacin and cotrimoxazole. Several limited antibiotics, such as ceftazidime-avibactam and minocycline, had unsatisfactory results in some patients. Among urinary antibiotics, nitrofurantoin showed efficacy against *E. coli* and *Klebsiella*, while nitrofurantoin and norfloxacin were effective against Pseudomonas and *Acinetobacter*. No urinary antibiotic shows effectiveness against Proteus (Table 3 - see PDF). A statistically significant variation was observed in the distribution of XDR isolates among different organisms (p = 0.00014), whereas the distribution of MDR (p = 0.754) and PDR (p = 0.448) did not demonstrate significant differences. A very strong positive correlation was identified between the total number of isolates and the total number of resistant isolates (r = 0.986, p = 0.000006). A moderate negative correlation was noted between MDR% and XDR% (r = -0.665, p = 0.072), while weak and statistically non-significant correlations were found between XDR% and PDR% (r = -0.384, p = 0.348) and between MDR% and PDR% (r = 0.317, p = 0.444) (Table 4 - see PDF).

## Discussion:

The increasing incidence of infections from multidrug-resistant (MDR) bacteria, or "superbugs," has become a critical concern in healthcare settings, significantly affecting morbidity and mortality rates [15, 16-17]. The improper use of antibiotics, increasing immuno-compromised individuals and insufficient infection control practices are critical contributors to the establishment and spread of multidrug-resistant bacteria [18]. The slow development of new antibiotics and the rapid emergence of resistant microbes exacerbate the issue. The WHO [7] states that expanding international trade and tourism further accelerates the global spread of drug-resistant diseases. Multiple studies have shown a rising prevalence of multidrug-resistant gram-negative bacterial infections, particularly among hospitalized patients [19, 20-21]. This study aimed to assess the prevalence of multidrug-resistant (MDR), extensively drug-resistant (XDR) and pan-drug-resistant (PDR) gram-negative bacteria, particularly in intensive care unit (ICU) settings. *Klebsiella spp*. was the most prevalent gram-negative bacterium in our study, including 277 of 792 isolates (34.9%), followed by *Escherichia coli* with 244 of 792 isolates (30.8%) and *Pseudomonas spp*. with 148 of 792 isolates (18.6%). These findings align with the studies conducted by Agyepong *et al.* [22] (24.5%), Basak *et al.* [23] (35%) and Folgori *et al.* [24] (67.6%), all of which commonly isolated *E. coli*. Our research revealed that 66.3% (792/1195) of the isolates had multidrug resistance, confirming the prevalent concerns regarding MDR diseases. *Klebsiella* spp. and *E. coli* were the primary multidrug-resistant pathogens in our study, with 21.2% (59/277) of *Klebsiella* and 22.5% (55/244) of *E. coli* demonstrating resistance to several antibiotics. Antibiotic susceptibility studies [13, 14, 15, 16-17] revealed that first- and second-line antibiotics, such as amoxiclav, ampicillin-sulbactam, cephalosporins, levofloxacin and gentamicin, demonstrated resistance rates below 60% for most illnesses. However, specialist antibiotics, including carbapenems, minocycline and piperacillin-tazobactam, had superior performance, with susceptibility rates ranging from 60% to 80%. Nitrofurantoin showed prolonged effectiveness against urinary isolates of *E. coli* and *Klebsiella* species. Our analysis indicated that commonly used first-line antibiotics, such as amoxiclav, ampicillin-sulbactam and levofloxacin, had low efficacy, showing less than 60% susceptibility to most gram-negative bacteria, including *Klebsiella*, *Escherichia coli* and *Pseudomonas*. In contrast, preserved antibiotics such as minocycline and carbapenems showed enhanced efficacy, 60-80% susceptibility and their capability to treat resistant infections. Significant disparities arise when comparing our findings with Saleem's study [25]. In Saleem's study [25], polymyxin-B had the most excellent efficacy as an antibiotic, with 92.1% susceptibility to *E. coli*, 100% to *Klebsiella* pneumoniae and 94.4% to *Pseudomonas* aeruginosa. Tigecycline had substantial efficacy in Saleem's research, showing 90.5% susceptibility to *E. coli* and 100% to *Klebsiella* pneumoniae, in stark contrast to our results, which revealed significantly lower susceptibility for *Klebsiella*. Saleem's research also showed that polymyxin B and tigecycline were almost completely effective against *Klebsiella* pneumoniae, with 100% susceptibility; however, our study found that susceptibility to these antibiotics was less than 60%. In Saleem's study, *Pseudomonas* aeruginosa showed 85.6% sensitivity to *colistin* and 94.4% to polymyxin-B; however, our results revealed a markedly moderate efficacy (60-80%) for both antibiotics.

Saleem's [25] research indicated that the most efficient antibiotics against all bacteria were polymyxin-B, tigecycline and fosfomycin, with polymyxin-B showing an overall efficacy of 91.9% and tigecycline displaying 88.1% effectiveness. This result corresponds with our findings, demonstrating that carbapenems and minocycline had enhanced susceptibility rates (60-80%), especially for resistant bacteria. Saleem's findings showed that polymyxin B had high efficacy against *Acinetobacter* (96.4%), followed by *colistin*(80%), supporting our findings, which suggested a similar susceptibility of *Acinetobacter spp*. to minocycline and carbapenems. In our study, XDR strains represented 26.4% (142/537) of ICU isolates, prevalence above the 16.2% observed in clinical wards. This result corresponds with findings from other studies, including those by Basak *et al.* [23] and Oliveria *et al.* [13], suggesting that MDR and XDR strains are more common in ICU settings due to prolonged hospital stays, frequent antibiotic use and invasive medical devices. Saleem's [25] research findings confirm the growing concern about MDR and XDR clinical isolates, similar to our results. In this study, 74.44% (606/814) of *E. coli* isolates exhibited multidrug resistance (MDR), whereas 0.24% (2/814) had extreme drug resistance (XDR). Of the *P. aeruginosa* isolates, 68.2% (310/454) were identified as multidrug-resistant (MDR), whereas 1.6% (5/454) was classed as highly drug-resistant (XDR). About 71.375% (192 of 269) of the *K. pneumoniae* strains were multidrug-resistant (MDR), while 0.37% (1 of 269) was extensively drug-resistant (XDR). The incidence of *S. aureus* exhibiting multidrug resistance (MDR) was 51.38% (445/866). In Saleem's study, 65.43% (1651/2523) of the isolates exhibited multidrug resistance (MDR), whereas 0.31% (8/2523) had extended drug resistance (XDR). This study failed to uncover PDR strains, unlike our findings, which documented PDR strains in *Acinetobacter spp*. and other organisms. The rising prevalence of XDR strains, particularly in ICU settings, underscores the need for improved infection control measures, antibiotic stewardship programs and the development of new pharmaceuticals. Despite the limited scope of our study, the emergence of PDR strains poses a significant threat to public health due to their resistance to practically all available antibiotics, including *colistin*, which is typically the last choice for treating MDR gram-negative infections.

## Conclusion:

We found a high frequency of antibiotic resistance in ICU isolates, primarily caused by *gram-negative bacilli* like *Klebsiella* and *E. coli*. Most first-line antibiotics showed insufficient sensitivity to these isolates, while second-line and reserved antibiotics showed moderate sensitivity.
